# Paralinguistic singing attribute recognition using supervised machine learning for describing the classical tenor solo singing voice in vocal pedagogy

**DOI:** 10.1186/s13636-022-00240-z

**Published:** 2022-04-15

**Authors:** Yanze Xu, Weiqing Wang, Huahua Cui, Mingyang Xu, Ming Li

**Affiliations:** 1 Data Science Research Center, Duke Kunshan University, Kunshan, China; 2 Advanced Computing East China Sub-Center, Suzhou, China

**Keywords:** Paralinguistic singing attribtue recognition, Vocal pedagogy, Music perception

## Abstract

Humans can recognize someone’s identity through their voice and describe the timbral phenomena of voices. Likewise, the singing voice also has timbral phenomena. In vocal pedagogy, vocal teachers listen and then describe the timbral phenomena of their student’s singing voice. In this study, in order to enable machines to describe the singing voice from the vocal pedagogy point of view, we perform a task called paralinguistic singing attribute recognition. To achieve this goal, we first construct and publish an open source dataset named Singing Voice Quality and Technique Database (SVQTD) for supervised learning. All the audio clips in SVQTD are downloaded from YouTube and processed by music source separation and silence detection. For annotation, seven paralinguistic singing attributes commonly used in vocal pedagogy are adopted as the labels. Furthermore, to explore the different supervised machine learning algorithm for classifying each paralinguistic singing attribute, we adopt three main frameworks, namely openSMILE features with support vector machine (SF-SVM), end-to-end deep learning (E2EDL), and deep embedding with support vector machine (DE-SVM). Our methods are based on existing frameworks commonly employed in other paralinguistic speech attribute recognition tasks. In SF-SVM, we separately use the feature set of the INTERSPEECH 2009 Challenge and that of the INTERSPEECH 2016 Challenge as the SVM classifier’s input. In E2EDL, the end-to-end framework separately utilizes the ResNet and transformer encoder as feature extractors. In particular, to handle two-dimensional spectrogram input for a transformer, we adopt a sliced multi-head self-attention (SMSA) mechanism. In the DE-SVM, we use the representation extracted from the E2EDL model as the input of the SVM classifier. Experimental results on SVQTD show no absolute winner between E2EDL and the DE-SVM, which means that the back-end SVM classifier with the representation learned by E2E as input does not necessarily improve the performance. However, the DE-SVM that utilizes the ResNet as the feature extractor achieves the best average UAR, with an average 16% improvement over that of the SF-SVM with INTERSPEECH’s hand-crafted feature set.

## Introduction

The term paralinguistic was first introduced by George L. Trager in the 1950s, relating to or denoting the nonlexical elements of communication of speech [1]. Paralinguistic attributes (properties) of speech play an important role in human communication. Much previous research works focus on speech emotion recognition [2–4]. Nowadays, due to the development of artificial intelligence, more and more new paralinguistic recognition tasks are proposed. Related competitions such as INTERSPEECH Computational Paralinguistic Challenges (ComParE) are held every year, releasing datasets and feature sets to help researchers worldwide address these tasks^1^. Nevertheless, there are few paralinguistic recognition describing the timbral phenomena of singing voices at present.

The timbre, also known as tonal quality or tonal color [5], was defined by “what distinguishes two sounds presented similarly and being equal in pitch, subjective duration, and loudness” by the American standard Association in 1960. Jody Kreiman discusses some limitations for different definitions of the timbre and claims that the definition of timbre should emphasize the interaction between signal and people [6]. Moreover, the timbral interaction is a complex process that can be affected by some aspects of the signal and stimulus, such as task type, stimulus characteristics, and stimulus context [6]. “Moreover, additional variability within a given listening task may be introduced by such listener characteristics as experience, memory, and attention.” The statements from Kreiman do not lift the veil of timbre, but it emphasizes that the timbral interaction is related to human perception and cognition, and the definition of timbre should not limit to a specific task type.

The timbral interaction about singing voice commonly happens in daily life, and related paralinguistic attributes indeed convey paralinguistic information among different groups of people. For example, people can describe a listening task by adjective terms; vocal pedagogists use vocal techniques to describe singing voices helping students develop a good voice; speech pathologists define and utilize some voice qualities to assess impaired voice.

Specifically, speech pathologists define a series of phonetic symbols as voice quality symbols (VOQS) for describing impaired speech voice [7] and perform auditory perceptual judgment based on ordinal scales of voice qualities to assess disordered voice [8, 9]. Furthermore, some acoustic researchers utilize perceptual dissimilarity coefficients between different pairs of single sustained vowels further to build the timbral space for performing timbral analysis [10–12]. Moreover, some vocal pedagogists and music theorists who have rich listening and singing experience use vocal techniques to describe singing voice [13]. In particular, some vocal techniques are metaphors to help students better understand and develop a good voice [14].

### Purpose and motivation

Regardless of confusing categories of paralinguistic attributes about voice qualities, metaphors, vocal techniques, and adjective terms, which are used in different domains for different purposes, our purpose is to let machines describe classical tenor singing voice using a set of singing-related paralinguistic attributes like a music expert. Since there are some music theories about describing singing voice systematically, some vocal pedagogists also use related terms to help students develop their voices in practice, which motivates us to select paralinguistic attributes mainly from vocal pedagogy, called paralinguistic singing attributes, to describe the classical tenor singing voice. Moreover, due to the rapid development of supervised machine learning and the increasing availability of data, which let us achieve our goal by performing the task called paralinguistic singing attribute recognition that recognizes or assesses certain singing attributes for singing voices extracted from classical tenor singing performances. Furthermore, nowadays, specific paralinguistic recognition tasks develop rapidly by designing different supervised machine learning algorithms, which motivates us to better address paralinguistic singing attribute recognition by exploring different machine learning frameworks.

### Related work

In music theory, there is some research systematically describing singing voice using different paralinguistic singing attributes based on different verbal scales [13, 15–17]. Specifically, David Blake offers four Booleans for adjective terms to describe the timbral phenomena in rock music—full, distorted, homogeneous, and digestible [15]. Heidemann builds a system using vocal techniques to describe the singing voice in pop music [13]. Wayne Slawson claims that the singing voices of sustained vowels can be described by four attributes—openness, acuteness, laxness, and smallness [16]. In particular, these four attributes should be further rated through the timbre space, which is a two-dimensional Cartesian coordinate system designed based on the vowel phonation [16].

Not only just describing in a systematic way, but there is some work further analyzing timbral phenomena by spectrum [18, 19]. In particular, Robert Cogan designs thirteen antonym pairs of adjective terms as oppositions (binary scale) which can be used to describe a wide range of repertoire [18]. Megan L. Lavengood uses different oppositions that are developed from cogon’s to perform spectral analysis for instrumental tones [19].

Besides, some researchers perform timbral analysis using dissimilarity matrix that collected by comparing dissimilarity coefficient of pairwise stimulus [10, 12]. And the dissimilarity matrix can be mapped into a visualized timbre space based on multidimensional scaling (MDS) algorithms. In particular, Brendan OConnor performs MDS for dissimilarity matrix of sustained vowels and further analyzes it by class averaging and clustering techniques [12]. For orchestral instrument tones, TM Elliott simultaneously performs MDS on dissimilarity matrix to generate MDS timbre space and performs discriminant function analysis (DFA) for semantic timbre space based on verbal scales of sixteen adjective descriptors [10]. Furthermore, Elliott rotates MDS timbre space to DFA results from semantic timbre space and assigns semantic ratings to MDS timbre space and then finds which descriptors in semantic timbre space combine to organize instrumental tones along the primary MDS dimension by bivariate linear regression.

Regardless of analysis, our task is recognition. And there is some research respectively performing tasks named vocal technique recognition and voice quality assessment using deep learning technique [20, 21]. Specifically, Wilkins et al. perform vocal technique recognition and publicize their dataset which is called VocalSet [20]. The VocalSet contains audio clips of singers vocalizing a range of pitches and sustained vowels. These clips are annotated by the different paralinguistic singing attributes such as straight, belt, breathy, fry, and vibrato. However, the singing voice can be described better using more terms based on existing music theories. Zwan performs voice quality assessment for singing vowels from a music expert point of view by supervised machine learning [21]. Unfortunately, the dataset is unavailable, and the singing attributes describing singing voices are unknown in their work. Furthermore, there are some other similar datasets that can not be accessed [22, 23].

To better describe classical tenor singing voice from a music expert perspective, it is necessary to select more paralinguistic singing attributes and then construct a dataset for supervised learning. Heidemann includes chest and head voice into her system to describe the pop singing voice [13]. These two terms and some other corresponding terms (e.g., chest register, chest resonance, head register, head resonance) are used in not only the teaching of western singing but music theater singing and pop singing [13, 24, 25]. Therefore, the chest and head resonance are the first two paralinguistic singing attributes that are selected.

Moreover, vowel phonation is related to singing quality. Specifically, western singers need to open their throats by mixed registration with certain centralized vowels for both sounding smoothly and singing loudly [26]. More evidence can be found since teachers let their students sing front or back metaphorically by using front placement and back placement singing techniques, which are related to the phonation of front and back vowels [27]. Furthermore, Slawson also borrows vowel phonation knowledge and sets openness as one dimension of the timbre space for subjectively quantifying the singing voice [16]. Therefore, we borrow front placement singing, back placement singing, and open throat as three paralinguistic singing attributes to describe singing voice. We select seven paralinguistic singing attributes and set different scales for them to describe classical tenor singing voice professionally. More information about these singing attributes can be found in Section 3.

In this work, we construct a publicly open classical tenor singing dataset called Singing Voice Quality and Technique Database (SVQTD)^2^. There are 4000 vocal solo segments with 4–20 s long. Each vocal segment is labeled by seven paralinguistic singing attributes based on different scales. To get vocal segments for labeling, we firstly download hundreds of audios from YouTube. And then, the vocal tracks of these audios are extracted from audios using music source separation. Furthermore, we perform energy-based silence detection to partition vocal tracks to vocal segments. Annotators who have studied classical singing in music department for at least 3 years are qualified for labeling vocal segments. This dataset can support subsequent research of performing supervised machine learning for paralinguistic singing attribute recognition.

The architecture of our supervised learning methods generally consists of two modules: a front-end processing unit that extracts the appropriate features from the available data and a back-end classifier that decides the paralinguistic attributes of the utterance. Depending on this architecture, we further implement three kinds of frameworks for addressing paralinguistic singing attribute recognition. And these three frameworks are already used in other paralinguistic recognition tasks:

- The first framework is to extract hand-crafted acoustic feature sets, e.g., OPENSMILE features [28, 29], at the front-end as the input of the back-end traditional classifier. Traditional classifiers are mathematical models such as support vector machine (SVM) [30, 31], *k*-nearest neighbors (KNN) [32–35], hidden Markov model (HMM) [30, 36, 37], and Gaussian mixture model (GMM) [32, 35, 38].
- The second framework directly trains an end-to-end neural network in which includes both a front-end processing unit and a back-end classifier. In particular, Trigeorgis proposes a convolutional nneural network-long short-term memory network (CNN-LSTM)-based model that handles the time-domain signal for the prediction of spontaneous and natural emotions [39]. Koike compares pre-trained audio neural network (PANN) to ResNet with and without pre-training on the COMPARE 2020 Challenge Mask Sub-Challenge [40]. Wilkins uses the time-delay neural network (TDNN), which is popular in speaker recognition, for the vocal technique recognition task on VocalSet [20].
- The third framework utilize robust feature representation from end-to-end neural network as the input of the back-end classifier. In particular, the representations are embeddings extracted usually from last several fully connected layers of the nerual network. This kind of framework performs well in paralinguistic recognition tasks with limited training data. Specifically, Amiriparian extracts spectrograms, which they pass into deep convolutional neural networks (AlexNet and VGG19) and then use the activations as the input of the SVM classifier for the INTERSPEECH ComParE 2017 Snoring sub-challenge [41]. Wu [42] explores both spectrograms and log-mel spectrograms as the input for deep convolutional neural networks (ResNet, Inception, and DenseNet) to extract representations; these representations are then used as the input of the SVM classifier for the INTERSPEECH ComParE 2019 orca activity and continuous sleepiness tasks.

Even though the popularity of end-to-end systems has increased tremendously, there is no absolute winner between traditional classifiers with hand-crafted feature sets and deep learning-based methods with limited training data [43]. Therefore, to better explore paralinguistic singing attribute recognition on SVQTD, we adopt all of these three aforementioned frameworks.

For the detailed implementation of each framework, we repectively explore using the INTERSPEECH 2009 Challenge’s feature set [28] and the INTERSPEECH 2016 Challenge’s feature set [29] as input, followed by SVM classifiers with the linear kernel. Each classifier is trained separately for each paralinguistic singing attribute. We abbreviate the first framework as SF-SVM (openSMILE features with support vector machine). Moreover, in the second end-to-end deep learning framework (D2EDL), the input of feature extractors is the spectrogram, and we separately explore the ResNet and the transformer as feature extractors. Each end-to-end model is trained for each paralinguistic singing attribute. Finally, for the third deep embedding with support vector machine (DE-SVM) framework, each end-to-end model in D2EDL is used to extract representation as to the input of the corresponding SVM classifier.

Moreover, a challenge for using the transformer is that the self-attention module of the transformer encoder brings a high computational cost for long sequential input, even though this module, as a main characteristic of the transformer, leads to good performance [44]. Compared with one-dimensional sequential input, the input of E2EDL is the two-dimensional spectrogram. To reduce the computational cost, Dosovitskiy calculates attention scores for each patch instead of for each pixel of an image [45]. Inspired by Dosovitskiy’s idea, we build a sliced multi-head self-attention module that slices the spectral input to patches to use the transformer in our paralinguistic singing attributes recognition with lower computational cost.

### Our contribution

The contributions from this work are as follows:

- Firstly, we select seven paralinguistic singing attributes that are helpful for beginners to learn classical singing and set label scales for them. Moreover, we construct SVQTD, which is a free and open classical tenor singing dataset for supervised learning studies. And to get sentence-level vocal segments of real singing performances, a certain production pipeline based on music source separation and silence detection techniques is proposed.
- Secondly, we explore three supervised machine frameworks in this task. Specifically, the ResNet and Transformer are separately explored as the feature extractor for the end-to-end method. Our result shows the DE-SVM framework based on ResNet achieves the highest UAR (unweighted average recall) metric for all paralinguistic singing attributes in average.

This paper’s remainder is as follows: Section 2 gives a description of SVQTD and its production pipeline. In Section 3, we introduce all the paralinguistic singing attributes selected in SVQTD and the label scale for each of them. In Section 4, we describe three supervised machine learning frameworks for paralinguistic singing attribute recognition in detail. The experimental setup, result, and conclusion are separately presented in Section 5, Section 6,and Section 7, respectively.

## Dataset description and data pre-processing pipeline

### Dataset description

**Fig. 1:**
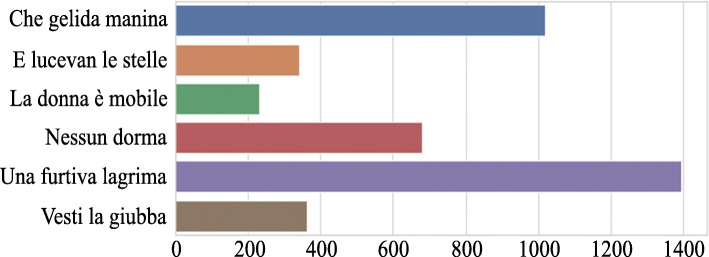
The number of vocal segments of each aria in SVQTD

Singing Voice Quality and Technique Database (SVQTD) is a classical tenor singing dataset collected from YouTube for performing paralinguistic singing attribute recognition tasks.^3^ In the SVQTD, there are nearly 4000 vocal solo segments with 4–20 s long, totaling 10.7 h. They are partitioned from four hundred audios downloaded from YouTube by searching the names of six famous tenor arias. The number of vocal segments to each aria is shown in Fig. 1. Furthermore, each segment is labeled on seven verbal scales corresponding to seven paralinguistic singing attributes. Table 1 shows the class number of each paralinguistic singing attribute.

**Table 1:** Number of vocal segments in each class of each paralinguistic singing attribute in SVQTD

Attributes	*C*_1_	*C*_2_	*C*_3_	*C*_4_	*C*_num_
Head resonance	101	804	2341	786	4
Chest resonance	191	967	2435	439	4
Open throat	2757	845	366	64	4
Roughness	3552	480	N/A	N/A	2
Vibrato	1052	2845	135	N/A	3
Front placement singing	1052	2845	135	N/A	3
Back placement singing	3157	724	151	N/A	3

### Data pre-processing pipeline

SVQTD is made by multi-stage approaches, starting from downloading YouTube videos. We use below pipeline to obtain thousands of labeled vocal segments.

#### Stage 1

Download videos from YouTube. The top 100 amateur videos of six famous tenor arias are retrieved from YouTube. To obtain the videos from singers who have different singing skills and levels of expertise, the words “cover|student|amateur” are appended to the song name to search for videos. All of the downloaded videos are converted to 16 kHz sampled waveform audio files (WAV). Moreover, we manually remove audios that are too noisy, non-solo, and repetitive. Note that we keep audios sang by the same singer but performed at different times. The reason is that singers may have different voice qualities and use different vocal techniques at different career stages.

#### Stage 2

Extract vocal tracks using end-to-end music source separation model. Music source separation is the task of decomposing music into its constitutive components, e.g., yielding separated stems for the vocals, bass, and drums. In recent years, end-to-end models including Open-Umix [46], spleeter [47], and Demucus [48] performs well on this task. And the performance between these models is compared in [47]. In this stage, the spleeter are used to extract vocal track from audios with accompaniment since we only focus on the timbral interaction between human listeners and singing voices.

#### Stage 3

Partition each vocal track to segments using energy-based silence detection. A short audio clip should be treated as silence if its energy is lower than a preset energy threshold. By adjusting the preset energy threshold, we can control the amounts of unsilence segments obtained from a vocal track. To make sure each segment obtained is between 4 and 20 s, we rerun this algorithm with a larger energy threshold on the segment that is longer than 20 s, and if a segment is shorter than 4 s, we will concatenate it with adjacent segments.

#### Stage 4

Subjectively label vocal segments for seven paralinguistic singing attributes are mentioned in Table 1. Seven annotators who have studied classical singing for more than 3 years in the music department of a college are recruited to annotate the vocal segments based on the labeling criteria defined in Section 3. Before annotating, they went through a 10-h training process to ensure that they were familiar with the paralinguistic singing attributes and have a relatively consistent understanding of how to label these data. Specifically, the 10-h training process includes 2 h for reviewing definitions of these attributes and 8 h for practicing labeling the data. In the reviewing process, we firstly introduce them to the paralinguistic singing attributes with label criteria. They discuss together and exemplify some singers based on the label criteria. In the pre-labeling process, they are informed to formally annotate the data. And a supervisor is responsible to check 100 labeled vocal segments for each annotator. This design is for avoiding carelessness, e.g., someone do not understand how to label or randomly label these data for saving time. Furthermore, the supervisor also should answer questions from annotators. During labeling, annotators found that the music source separation module may lead to degradation on some vocal segments. And the vocal segments that extremely interfere with the perceptual judgment are asked to remove from the dataset. Some typical bad samples caused by music source separation module are listed on the GitHub dataset page.

## The paralinguistic singing attributes and labeling criteria

As forementioned, we select chest resonance and head resonance as two paralinguistic singing attributes to describe classical singing voice. Besides, in vocal pedagogy, chest and head are also used for the noun adjunct of both the voice and the register. To better introduce chest and head resonance, we will begin with introducing the vocal register and the vocal resonance in Section 3.1. Since the vocal register is also defined in speech pathology, we will also introduce it and then discuss why vocal registers from speech pathology are not suitable to describe classical tenor singing voices from a music perspective. In Sections 3.2 to 3.8, we separately introduce seven paralinguistic singing attributes chosen. For singing attributes without a consistent definition but represent a certain timbre to describe the singing voice, we describe our adopted labeling criteria from the vocal color point of view. Besides, we also provide some relevant background for these singing attributes about acoustics, physiology, and phonation.

**Fig. 2:**
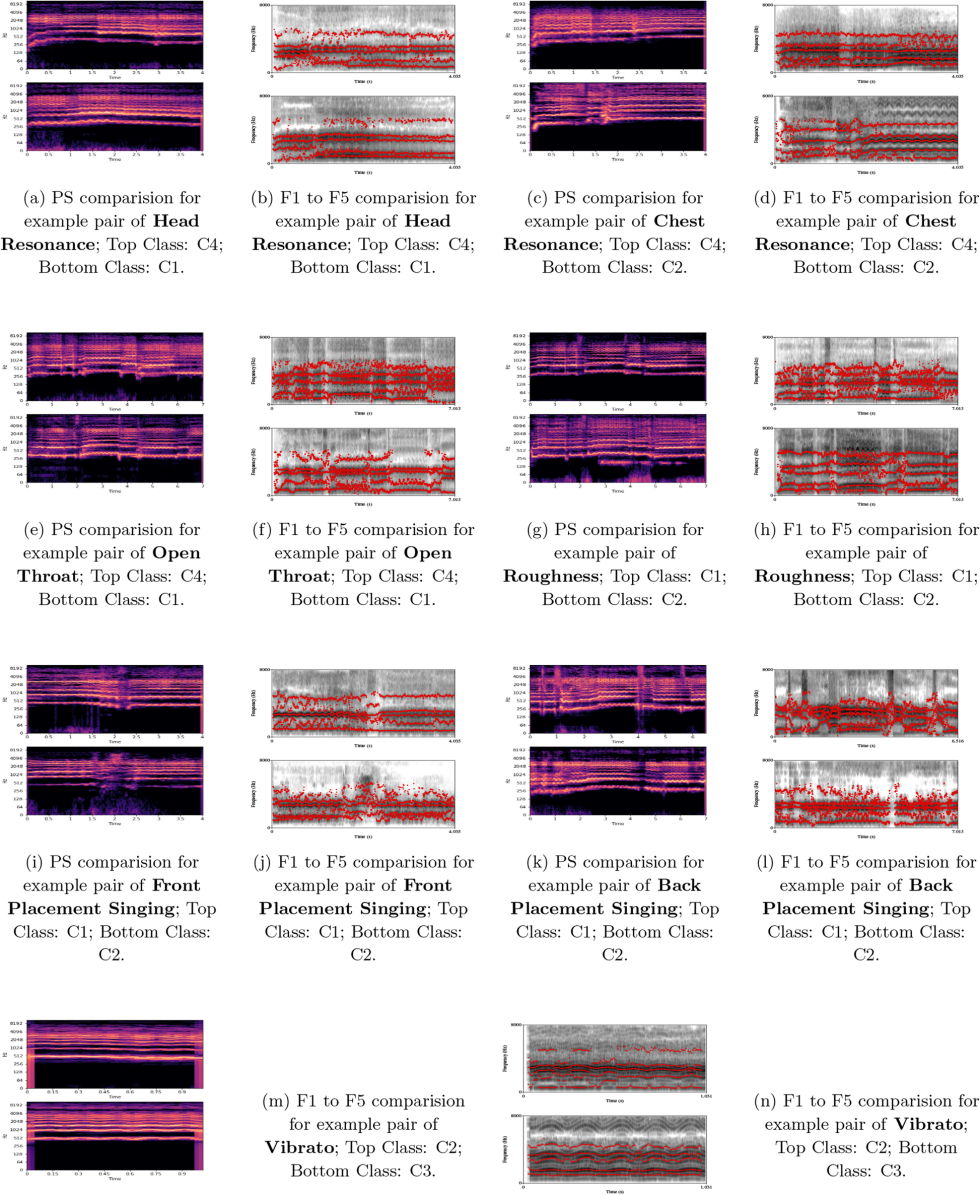
Visual comparison of example pairs of paralinguistic singing attributes. PS, wide-band power spectrogram; F#, Tte corresponding formants depicted on the narrow-band spectrogram

To better understand these attributes, we also present a pair of examples for each attribute and list them on the GitHub dataset page. The data visualizations of each example pair are presented in Fig. 2. Specifically, we transform them into both the wide-band power spectrogram with larger window length and the narrow-band spectrogram with narrow window length. The first five formants are also shown on the narrow-band spectrogram by using Praat [49].

### Vocal register and vocal resonance

In speech pathology, vocal registers arise from different vibratory patterns produced by the vocal cords. These registers include modal voice, vocal fry, falsetto, and the whistle register [50–52]. In particular, the modal register is the most common register in singing. A well-trained singer or speaker can phonate two octaves or more in the modal register. However, vocal registers from speech pathology are not suitable to describe or discriminate tenor singing voices. One of the reasons is that it is meaningless to say that a tenor singing voice is a modal voice since most of the tenors sing classical songs with a modal voice. Moreover, vocal fry and whistle register is not practical in classical singing.

In vocal pedagogy, the first recorded mention of the chest and head voice was around the thirteenth century [53]. During the bel canto period, chest and head voices were redefined as the lowest and the highest of three vocal registers: the chest, passaggio, and head registers. It is still used in the teaching of bel canto today [24]. The chest and head voice may refer to different meanings: (i) a type of vocal register, (ii) a vocal resonance area, and (iii) a specific vocal timbre [51].

Rigorously, the chest or head can not produce voice, it is related to vibratory sensations in the chest or head. Moreover, “since all registers originate in laryngeal function, it is meaningless to speak of registers being produced in the chest or head” [51]. McKinney states that the vibratory sensation is actually the resonance phenomenon, and he defined vocal resonance as “the process by which the basic product of phonation is enhanced in timbre and/or intensity by the air-filled cavities through which it passes on its way to the outside air” [51]. In summary, to describe the singing voice, no matter chest voice, register, or resonance, all of them are terms related to specific resonance phenomena and can also be used to represent certain timbre phenomena in vocal pedagogy.

However, it is hard to further describe the certain vocal timbre that specific paralinguistic singing attributes represent by some adjective terms. If using singing attributes for different timbral interaction situations, even music theorists use different adjective terms for the same term. For example, J Stark describes chest voice using qualities such as dark, covered, and full [24]. However, in his work, he mentions Lodovico Zacconi, who prefers bright and ringing chest voice, uses stinging and biting quality for describing chest voice [24]. Moreover, in the work about female chest voice by JL LoVetri, she mentioned that “there traditionally has been debate among pedagogues as to whether or not chest register is responsible for the deeper, darker color of the sound or for the ‘edge’ or brilliartce in the resonance” [54]. Therefore, it is hard to give a fully correct description of these attributes for every situation. And this is why we focus on the tenor singing voices instead of describing the timbral phenomena of all classical singing voices, which means we think it will be easier to make a consensus on a narrower extent after performing training before annotation.

### Chest resonance

The chest resonance represents the resonance area in the chest, and the resonance phenomena give the singer a vibratory sensation in the chest [51]. Chest resonance is related to a darker, deeper tone coloring [54, 55]. Acoustic research shows that chest resonance has a higher response to open vowels [56]. During labeling, annotators assess each vocal segment and subjectively assess the extensity of chest resonance by a 4-class ordinal scale. For reference only, the 4 for a group of singing voices that are darkest, strongest, and with strongest vibratory sensation compared with other singing voices in the dataset. Level 1 for a contrary situation. The judgment process is based on listeners’ music cognition and perception.

### Head resonance

The head resonance represents the resonance area in the head, and the resonance phenomena give the singer a vibratory sensation in the head [51]. The head resonance is primarily for softer singing [57]. From the physiological perspective, singers raise their soft palate and narrow the epilaryngeal inlet [57]. And as the pitch rises, the vocal folds gradually tense, and only the thinned outer layers of the vocal folds can vibrate [13]. Acoustic research shows that head resonance is related to the singer’s formant [58]. During labeling, the annotators are told to subjectively rate the 4-class ordinal scale of head voice from 1 to 4 for the vocal segments. For reference only, 4 for a group of singing voices with best head register technique which means smoothest and richest quality compared with other singing voices in the dataset. And level 1 for a contrary situation.

### Front placement singing

The subjective judgment of chest and head resonance can roughly describe many singing voices. However, other resonators have been used to describe the singing voices in vocal pedagogy. One of the resonators is mask/nasal resonance, which is related to nasal quality [51]. Debertin proposes a method for perceptual judgments of nasal resonance of singing [59]. Wooldridge shows that nasal resonance is not being utilized as resonators if classical singers block nose with cotton [60]. To avoid nasal quality, the rise of velum, closure of velopharyngeal port can reduce airflow through the nasal passage [61]. Since nasal quality is difficult to subjectively judge and there are nasal consonants during singing, we focus on the vocal technique, the forward placement singing, or named mask singing, which has an obvious nasal quality [27, 62]. Researchers utilize low tone to high tone ratio (VLHR) acoustic features to evaluate nasal quality [63]. And VLHR is used for the evaluation of hypernasality in vowels [64]. During labeling, listeners need to judge whether the singing segment is mask singing/forward placement singing with an obvious nasal quality based on a binary scale, 0 for without, 1 for always with, 2 for sometimes with.

### Back placement singing

Back placement is popular in opera performances. However, some negative adjectives, such as swallowing, are used to criticize the excessive back placement singing [51, 65]. The “extreme” back placement does not help the resonance and often has excessive muscle tension [66]. Compared with back placement singing, Vurma and Ross perform spectral analysis, which shows that front placement singing is not only with higher frequencies of the first and second formants but also with the higher frequency and level of the singer’s formant [62]. Wyllys performs acoustic and articulatory research on both forward placement and back placement singing [65]. During labeling, annotators need to subjectively judge whether the singing segment is “extreme” back placement singing which is characterized by uncomfortable swallowing quality based on a binary scale, 0 for without, 1 for always with, 2 for sometimes with.

### Open throat

In Western singing, teachers train students to open their throat by mixed registration with certain centralized vowels to sing loudly and sound smoothly [26]. Slawson also borrows vowel phonation knowledge and sets openness as one dimension of the timbre space for subjectively quantifying the singing voice [16]. The open throat scale ranges from 1 to 4. For reference only, level 1 for a group of singing voices that feel extremely uncomfortable, strained, and narrow, and level 4 for singers who are very good at performing mix registration using centralized vowels that make listeners feel the obvious open quality.

### Roughness

In speech pathology, roughness is usually rated on an ordinal scale in multiple dysphonic vocal quality assessment protocols, e.g., breathiness and hoarseness (RBH), consensus auditory perceptual evaluation voice (CAPE-V), and grade, roughness, breathless, asthenia, strain (GRBAS) [9]. Singing in the wrong way may produce a rough, raspy voice that may harm the singer’s voice. And classical singers do avoid making their voice sound raspy. During labeling, annotators pay attention to judge whether there is apparent roughness in the vocal segment, 0 for without, 1 for with.

### Vibrato

Singers from various musical genres use vibrato while singing. There are good vibrato and bad vibrato (tremolo and wobble), and the tension in the breathing, neck, or vocal mechanisms may cause faulty vibrato [67]. There are good vibrato and bad vibrato (tremolo and wobble) [67]. As mentioned in Section 1.3, frequency and amplitude variations are important acoustic parameters used to judge good vibrato and bad vibrato [68]. Wobble has a wider pitch fluctuation and a slower frequency than good vibrato, while tremolo has a narrower pitch fluctuation and faster frequency than good vibrato [69]. Vibrato can be used in the singing evaluation systems [70] and in singer classification [71]. During labeling, the annotators should focus on the vibrato of vocal segments, rating segments as 0 for having no vibrato, 1 for having good vibrato, or 2 for having bad vibrato.

## Supervised machine learning frameworks for paralinguistic singing attribute recognition

SVQTD is obtained by the aforementioned data pre-processing and annotation with 4000 labeled vocal segments. Hence, we use SVQTD to perform the paralinguistic singing attribute recognition task by supervised machine learning. In this section, we first formulate the problem in Section 4.1; then the adopted three methods (SF-SVM, E2EDL, and DE-SVM) are described with details in Sections 4.2, 4.3, and 4.4, respectively.

### Problem definition

We assume a set of *n* training vocal segments $\mathbb {S} = \{S_{i}\}^{n}_{i=1}$ with corresponding labels $\mathbb {Y} = \{Y_{i}\}_{i=1}^ n$. *Y*_*i*_ is a set of *n*_attr_ labels for the corresponding paralinguistic singing attributes, $Y_{i} = \{y_{i}^{j}\}_{j=1}^{n_{\text {attr}}}$, where $y_{i}^{j} \in [1,\cdots n_{\text {class}}^{j}]$ and $n_{\text {class}}^{j}$ is the number of classes of each paralinguistic singing attribute, as defined in the label criteria. Our paralinguistic singing attributes recognition task contains *n*_attr_ classification subtasks corresponding to *n*_attr_ paralinguistic singing attributes. We implement three frameworks to explore better ways of solving each subtask.

### The SF-SVM framework

**Fig. 3:**
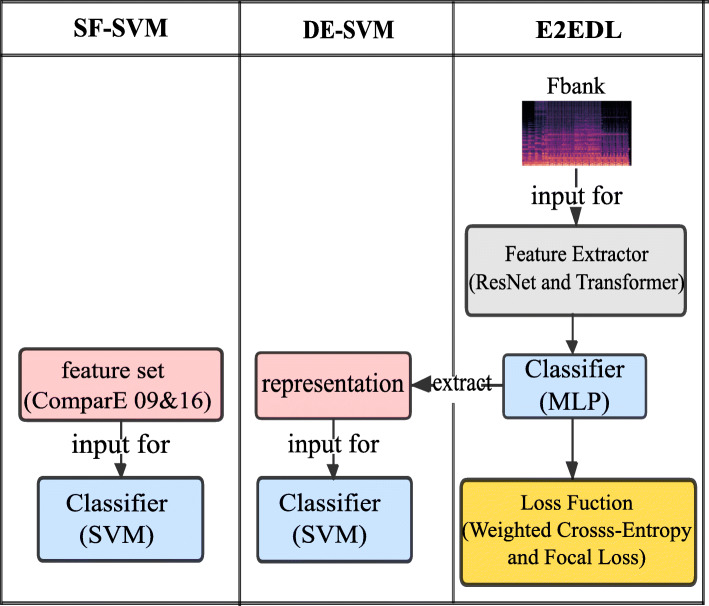
The proposed three frameworks

As mentioned in Section 1.2, a common framework for solving paralinguistic attribute recognition tasks utilizes a set of acoustic and prosodic features as the input of the traditional machine learning classifier [30, 32, 72]. Referring to this framework, we design the SF-SVM framework as shown in the left column of Fig. 3. Specifically, for the front-end feature extraction, we extract both the feature set of INTERSPEECH 2009 emotion challenge and that of INTERSPEECH 2016 challenge [73] (Table 2). And we train the SVM classifier with linear kernel as the back-end classifier for each classification subtask with two feature set as input.

**Table 2:** Features in the INTERSPEECH 2009 emotion challenge feature set

LLD (16*2)	Functionals (12)
(*Δ*) ZCR	Mean
(*Δ*) RMS energy	Standard deviation
(*Δ*) F0	Kurtosis, skewness
(*Δ*) HNR	Extremes: value, rel. position, range
(*Δ*) MFCC 1-12	Linear regression: offset, slope, MSE

#### Feature set

The INTERSPEECH 2009 emotion challenge feature set (ComparE09) includes 16 low-level descriptors: zero-crossing rate (ZCR) from the time signal, root mean square (RMS) frame energy, pitch frequency (normalized to 500 Hz), harmonics-to-noise ratio (HNR) by autocorrelation function, and 12 dimensional mel-frequency cepstral coefficients (MFCC) features. The delta coefficients and various kinds of functionals, e.g., mean, standard deviation, kurtosis, skewness, minimum and maximum values, relative position, and range as well as two linear regression coefficients with their mean square error (MSE) are applied to generate 16×2×12=384 dimensional utterance level feature vectors. Moreover, the INTERSPEECH 2016 challenge feature set includes 6373 static features which is more powerful and comprehensive. Its fully description can be found in [74].

### The E2EDL framework

The right column in Fig. 3 presents our E2EDL framework that uses the log-mel spectrogram (Fbank) as input. The network architecture consists of a front-end feature extractor and a back-end classifier. Specifically, we use a multilayer perceptron (MLP) as the back-end classifier. The Resnet and transformer encoder serve as the feature extractors; thus, we implement two types of end-to-end frameworks and experiment with both of them. For the transformer’s encoder, we transform the multi-head attention (MSA) module into a slice multi-head self-attention (SMSA) to deal with the log-mel spectrogram input. Here, we first introduce the standard transformer’s encoder for automatic speech recognition (ASR) (4.3.1). The transformer with SAMA as the feature extractor is introduced in Section 4.3.2. Finally, the details of the model construction, the loss function, and the evaluation metrics are presented in Section 4.3.3.

#### The standard transformer encoder

**Fig. 4:**
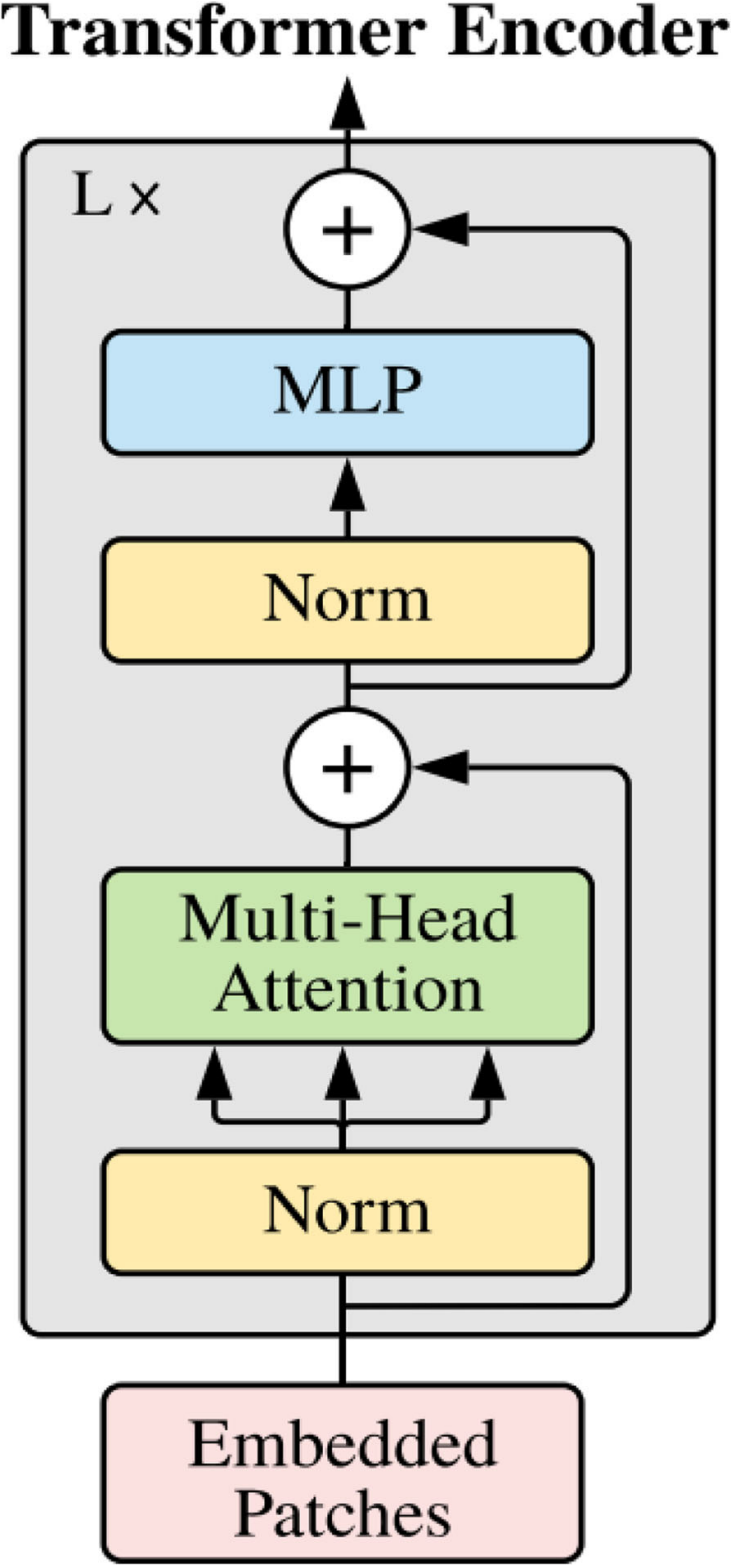
The standard transformer encoder architecture

The standard transformer encoder architecture proposed in [44] includes multi-head self-attention (MSA) and a multilayer perceptron (MLP), with layernorm (LN) [75] and skip connection [76] operations in between (Fig. 4). The main module, multi-head self-attention, is derived from self-attention (SA).

Equations (1), (2), and (3) present the calculation of self-attention. Specifically, for an input Fbank sequence $\mathbf {x} \in \mathbb {R}^{T\times F}$, *T* is the length of the time sequence, and *F* is the number of Mel filters. The $\mathbf {Q, K, V} \in \mathbb {R}^{T\times F}$ is the linear projection of **x**. The attention scores **A** are calculated by the multiplication of **Q** and **K** representations.

1 $$\begin{array}{*{20}l} Q, K, V = x[U_{q};U_{k};U_{v}]  \end{array} $$

2 $$\begin{array}{*{20}l} A=\text{softmax}(QK^{T}/\sqrt{C}),  \end{array} $$

3 $$\begin{array}{*{20}l} SA(x)=AV.  \end{array} $$

Here, $U_{q}, U_{k}, U_{v} \in \mathbb {R}^{F\times F}$. $1/\sqrt {C}$ is a regulating term to avoid a large inner product as well as gradient vanishing.

MSA is an extension of SA, in which *h* self-attention operations, called “heads,” are connected in parallel and project their concatenated outputs. To keep the dimension consistent when changing *h*, another linear projection by $U_{msa}, U_{msa} \in \mathbb {R}^{kF\times F}$ should be calculated as shown in Eq. (4).

4 $$\begin{array}{@{}rcl@{}} \text{MSA}(x)=[SA_{1}(x);\dots; SA_{h}(x);]U_{\text{msa}}.  \end{array} $$

#### Transformer encoder with the sliced multi-head attention

**Fig. 5:**
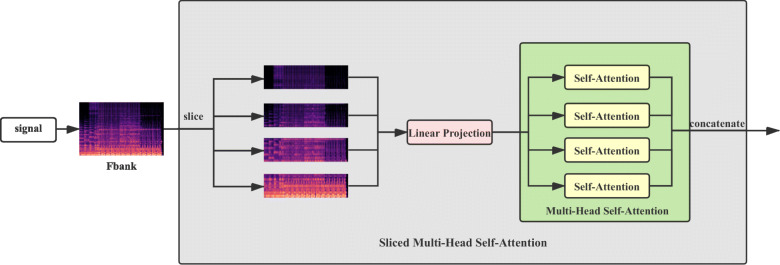
The architecture of our SAMA model

The standard transformer encoder used in automatic speech recognition (ASR) is for handling one-dimensional sequences. Thus, we modified the standard transformer encoder as our feature extractor so that it can handle two-dimensional spectrogram input. Specifically, the original MSA is replaced by the sliced multi-head self-attention (SMSA). The architecture of the SMSA is shown in Fig. 5. The log-mel spectrogram is divided into *k* slices, transformed by linear projection, and then fed into *h* “heads” of multi-head self-attention. Figure 5 illustrates when *k* equals *h*, and The calculation is formulated in Eqs. (5) and (6).

5 $$\begin{array}{*{20}l} x = [S_{1}; S_{2}; \dots S_{k-1}; S_{k}]  \end{array} $$

6 $$\begin{array}{*{20}l} \text{SMSA}(x) = [SA_{1}(S_{1}); \dots ;SA_{h}(k)]  \end{array} $$

#### Model construction

There are two types of end-to-end models: one is based on the ResNet, and the other one is based on the transformer encoder with SAMA. To construct the former, we utilize the ResNet34 [76]. We present the model architecture of the latter in Table 3.

**Table 3:** The details of the proposed transformer encoder model. *TE* transformer encoder, *SMSA* sliced multi-head self-attention, *MSA* multi-head self-attention, *MLP* multi-layer perceptron, *FC* fully connected, *MP* global max pooling

Layer			Parameters	Output
Extractor *TE*×3	SAMA	Slice	*k*=4	*T*_dim_×32
		*FC*×*k*	[1×1,32]	*T*_dim_×32
		MSA	*h*=4	*T*_dim_×32
	MLP	Cat	*k*=4	*T*_dim_×128
		FC	[1×1,128]	*T*_dim_×128
		FC	[1×1,512]	*T*_dim_×512
		FC	[1×1,128]	*T*_dim_×128
		GMP	[*T*_dim_×1,1]	1×128
Classifier	MLP	FC	[1×1,64]	1×64
		FC	[1×1,*C*_num_]	1×*C*_num_

For the loss function, we first train the neural network using cross-entropy. However, the neural network is inclined to predict all the samples as the majority class, which is caused by the imbalanced classes in SVQTD. Rosenberg presents a method to assign the importance weight for each class in the SVM classifier [77]. On Interspeech 2009 Emotion Challenge tasks, their importance weighting for SVM achieves the best unweighted average recall (UAR) compared with other sampling techniques, such as the oversampling and undersampling [77]. Moreover, importance weighting is also used on the weighted cross-entropy loss for the end-to-end method. Lin proposes the focal loss to address the imbalanced problem for dense object detection [78]. Therefore, we use both the weight cross-entropy and the focal loss simultaneously as the loss function for training our end-to-end models, and the UAR is also served as an evaluation metric in our work.

### The DE-SVM framework

The DE-SVM framework is shown in the middle column of Fig. 3. The representation is used to replace the hand-crafted feature set as the input of the SVM clas- sifier. Specifically, we extract embedding representations from the penultimate layer of the MLP classifier of every end-to-end model that is trained for each classification subtask. Importance weighting is also used here for SVM training.

## Experimental setup

This section describes the experimental setup and design, covering feature extraction, data processing, data preparation, training, and evaluation.

### Features extraction

For SF-SVM, both the INTERSPEECH 2009 Emotion Challenge’s feature set and the INTERSPEECH 2016 Challenges’s feature set are extracted by OpenSMILE [28]. To the log-mel spectrogram input for the end-to-end model, we first compute the short-time Fourier transform (STFT), and then map the power spectrogram on the Mel-scale. The STFT is the 4096-point discrete Fourier transform using a Hanning window with 75% overlap between frames. Furthermore, the number of band-pass filters is 128.

### Data processing

For the music source separation in the dataset production pipeline, we use the two-stem model provided by spleeter^4^. Furthermore, we use the silence detection algorithm embedded in Pydub^5^.

### Data preparation

SVQTD is divided into training, validation, and testing subsets. We try our best to avoid data leaking, i.e., making sure that the vocal segments do not coexist in multiple subsets.

### Training and evaluation

The SVM classifier is trained by the scikit-learn toolkit [79]. For training the SVM classifier, we adopt the linear kernel, the balanced class weight, and grid searching the complexities of 1, 0.1, 0.001, 0.0001, 0.00001, 0.000001; the other parameters are set to default. The model that obtains the best unweighted average recall (UAR) in the validation set with specific parameters is used for evaluation. Furthermore, the end-to-end model is implemented and trained on PyTorch [80]. The model construction is detailed in Section 4.3.3. The focal loss’s parameter *γ* is set to 2, and class weights for the weighted cross-entropy loss are obtained by dividing the number of each class by the number of the minority class. We utilize Adam as the optimizer, with an initial learning rate of 0.0001 and 50 percentage dropout for the MLPs. During training, the batch size of the models based on Resnet is 4, and that based on the transformer is 8. Moreover, we employ an early stop strategy to halt the training process when the validation UAR does not improve for more than 10 epochs. Finally, the best UAR result on the validation set is used for evaluation. Note that the random seeds of both Pytorch and scikit-learn are fixed to avoid biased results.

## Result

To handle our paralinguistic singing attributes recognition task, we separately train the classifiers for seven attribute classification subtasks with different numbers of classes. Since we adopt two different network structures, the CNN-based ResNet and RNN-like transformer, the related frameworks of DE-SVM and E2EDL can be further divided. Since data from different labels are imbalanced, we use UAR that considers the recall percentage of each class equally as the metric. The UAR results of the 4-class, 3-class, and 2-class paralinguistic attribute classification subtasks are shown in Tables 4, 5, and 6, respectively.

**Table 4:** Different frameworks’ UAR results for classification subtasks of three 4-class paralinguistic singing attributes

	Unweighted average recall (UAR) [%]			
Frameworks	Chest resonance	Head resonance	Open throat	Average
SF-SVM (ComparE09)	34	37.21	28.1	33.10
SF-SVM (ComparE016)	38.7	34.34	**46.2**	39.74
E2EDL (ResNet)	44.39	37.33	28.8	36.84
E2EDL (Transformer)	41.54	37.68	29	36.07
DE-SVM (ResNet)	**46.63**	**44.43**	30.82	**40.63**
DE-SVM (Transformer)	42.17	40.26	22.58	35

**Table 5:** Different frameworks’ UAR result for classification subtasks of three 3-class paralinguistic singing attributes

	Unweighted average recall (UAR) [%]			
Frameworks	Front placement singing	Back placement singing	Vibrato	Average
SF-SVM (ComparE09)	31.87	34.91	35.52	34.10
SF-SVM (ComparE016)	33.7	33.76	42.84	36.77
E2E (ResNet)	**35.2**	36.2	41.89	37.76
E2E (Transformer)	33.6	**39.42**	38.97	37.33
DE-SVM (ResNet)	33.22	33.76	**47.02**	**38**
DE-SVM (Transformer)	30.61	36.71	43.67	37

**Table 6:** Different frameworks’ UAR result for binary classification subtask of roughness

	Unweighted average recall (UAR) [%]
Frameworks	Roughness
SF-SVM (ComparE09)	51.85
SF-SVM (ComparE016)	55.19
E2E (ResNet)	56.23
E2E (Transformer)	55.39
DE-SVM (ResNet)	**58.83**
DE-SVM (Transformer)	54.4

As shown in Table 4, the ResNet based DE-SVM system performs best on recognizing chest resonance and head resonance. For the recognition of the open throat, the ComparE16 feature set based SF-SVM approach performs better. We believe the reason might be that some features in the ComparE16 feature set contain discriminative information about the open throat attribute.

Table 5 shows the result of three 3-class classification subtasks. ResNet-based DE-SVM also achieves the highest average UAR percentage. However, the ResNet-based DE-SVM only performs best on the third subtask, the recognition of the Vibrato, and the best framework for the first two subtasks is E2EDL, which reflects that the use of the SVM classifier for representation does not necessarily improve performance in these subtasks. The ResNet-based E2EDL framework achieves the highest UAR for front placement singing, while the transformer-based E2EDL framework achieves the highest UAR for back placement singing. Furthermore, SF-SVM does not perform well here. Table 6 shows the results of binary classification.

In summary, ResNet-based DE-SVM has the best overall performance, which achieves the highest UAR on four subtasks. However, the subsequent SVM classifier for the representation learned by E2EDL does not necessarily improve the performance on top of E2EDL on all subtasks which might be affected by the difficulties of our task, the amount and quality of the training data, etc. Therefore, we need to try different frameworks and explore different neural networks’ feature extractors for classifying different paralinguistic singing attributes. In general, the SF-SVM framework has lower UAR compared to deep learning based methods. However, there is no absolute winner all tasks, since the ComparE016 feature set based SF-SVM approach achieves the highest UAR on the open throat task. In the future, we will collect a large-scale database and evaluate the proposed methods again.

It is worth noting that the classification accuracy is still quite low which is far away from real applications. But we believe that the proposed problem formulation, labeling criteria, data pre-processing pipeline, open-source database, and different machine learning frameworks would contribute to the research in the paralinguistic singing attribute recognition field.

## Future work

In the future, we aim to firstly improve our dataset production pipeline. In particular, it is necessary to further update the music source separation module with more powerful models to avoid quality degradation.

Secondly, it is needed to make more data for both robust training and better prevent data leaking.

- To prevent data leaking, our current method is firstly to avoid the same segment existing in any of the three sub-datasets, and then make sure that sub-datasets are respectively with segments from different arias. However, there is still a better way to prevent data leaking, which is to avoid segments of the same singer existing in other sub-datasets. Therefore, we will collect more audios with singer identity to better perform dataset splitting.
- For robust training with machine learning algorithms, it is necessary to get more judgment results from different music experts for each sample. With more data, we can perform more experiments to observe the linear correlation between data collected from different annotators. If the correlation between two experts is weak or moderate, it is hard to say which music expert is right, it will be interesting to further analyze embedding space to interpret this phenomenon.

## Conclusion

To enable a machine to describe a singing voice as a human in vocal pedagogy would and to help beginners in their vocal training, we propose the paralinguistic singing attribute recognition task. We construct a classical tenor singing dataset called SVQTD for exploring different supervised learning methods. We also propose a pipeline with music source separation and silence detection to pre-process the data; and introduces labeling criteria for each paralinguistic singing attribute.

For the supervised machine learning algorithms, we implement three frameworks, namely SF-SVM, E2EDL, and DE-SVM. Moreover, to use two-dimensional spectrograms as the input for the transformer, we modify the multi-head self-attention to a sliced version. Our experimental results show no absolute winner between E2EDL and DE-SVM, which means the subsequent SVM classifier for the deep embedding representation learned by E2EDL does not necessarily improve the performance for every single subtask. Moreover, the DE-SVM that utilizes the ResNet as the feature extractor achieves the best average UAR for majority attributes.

## Data Availability

https://hackerpeter1.github.io/SVQTD/.

## Abbreviations

SVQTD: Singing Voice Quality and Technique Database
SF-SVM: openSMILE features with support vector machine
E2EDL: End-to-end deep learning
DE-SVM: Deep embedding with support vector machine
SMSA: Sliced multi-head self-attention
ComParE: Computational paralinguistic challenges
COVID19: Coronavirus disease of 2019
VOQS: Voice quality symbols
MDS: Multidimensional scaling
DFA: Discriminant function analysis
SVM: Support vector machine
KNN: K-nearest neighbors
HMM: Hidden Markov model
GMM: Gaussian mixture model
CNN: Convolutional neural network
LSTM: Long short term memory network
PANN: Pre-trained audio neural network
TDNN: Time-delay neural network
ResNet: Residual neural network
UAR: Unweighted average recall
WAV: Waveform audio file
VLHR: Voice low tone to high tone ratio
RBH: Breathiness and hoarseness
CAPE-V: Consensus auditory perceptual evaluation voice
GRBAS: Grade, roughness, breathless, asthenia, strain
ZCR: Zero-crossing rate
RMS: Root mean square
HNR: Harmonics-to-noise ratio
MFCC: Mel-frequency Cepstral coefficients
MSE: Mean square error
MLP: Multilayer perceptron
MSA: Multi-head self attention
ASR: Automatic speech recognition
LN: Layernorm
STFT: Short-time fourier transform
RNN: Recurrent neural network

## Footnotes

1 http://www.compare.openaudio.eu/winners/

2 https://hackerpeter1.github.io/SVQTD/

3 https://hackerpeter1.github.io/SVQTD/

4 Spleeter, 2021, Available: https://github.com/deezer/spleeter

5 Pydub, 2021, Available: https://github.com/jiaaro/pydub
